# Experimental Characterisation and Finite Element Modelling of Polyamide-12 Fabricated via Multi Jet Fusion

**DOI:** 10.3390/polym14235258

**Published:** 2022-12-02

**Authors:** Kok Peng Marcian Lee, Mladenko Kajtaz

**Affiliations:** School of Engineering, RMIT University, P.O. Box 71, Bundoora, VIC 3083, Australia

**Keywords:** Multi Jet Fusion, polyamide 12, mechanical properties, finite element analysis

## Abstract

The HP Multi Jet Fusion (MJF) technology is a relatively recent addition to powder bed fusion additive manufacturing (AM) techniques. It differentiates itself from selective laser sintering (SLS) technology through the use of fusing and detailing agents to control part geometry, and the use of a planar infrared radiation (IR) source that sweeps over the powder bed to initiate the sintering process. Depending on the printing methodology, AM processes can introduce mechanical property anisotropy that is dependent on print orientation. In the case of MJF-fabricated parts, there is a general disagreement over the influence of print orientation on tensile mechanical properties in the literature. In this work, MJF-fabricated PA12 (AM PA12) is printed at various orientations and characterised in terms of tensile and compressive mechanical properties. The orientations have been selected to take into account the alignment of the IR source sweep direction to the test load. We observe that orientating parts towards the vertical direction for printing tends to favour enhanced tensile mechanical properties. The anisotropy in mechanical properties is attributed to more complete polymer powder fusion as a result of the increased number of IR source sweeps when parts are orientated towards the vertical direction. Both tensile and compressive stress–strain data were used as experimental data input for calibrating the Elastic–Plastic with combined hardening (EPC) material model in the commercial finite element analysis (FEA) package—Abaqus. We demonstrate that the EPC material is a suitable material model for the FEA of AM PA12.

## 1. Introduction

Additive manufacturing (AM), commonly referred to as 3D-printing, is a process for the fabrication of products through a layer-by-layer approach from virtual models [1,2]. These models are typically created either via acquired image data (e.g., 3D-scanning) or computer-aided design (CAD). The models are then processed by a 3D slicer software, which converts the model into a suitable toolpath file for the 3D printer. As parts are built by fusing the material together, AM processes can reduce waste, unlike the traditional approach of removing material from a billet [1]. Furthermore, the use of AM techniques enables the fabrication of highly complex designs that would have otherwise been too expensive or challenging to produce via other methods.

**Figure 1:**
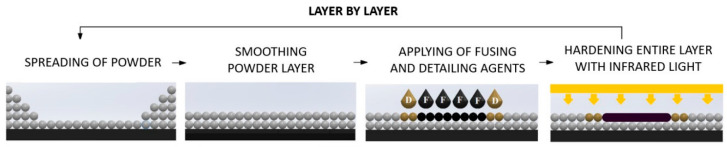
Schematic of the MJF fabrication process [4].

There are many approaches towards AM, including material extrusion, powder bed fusion, and vat photopolymerisation, each with their respective advantages and limitations [3]. The HP Multi Jet Fusion (MJF) technology is an example of powder bed fusion AM. The MJF process involves the deposition of the polymer powder, application of a fusing and detailing agent, followed by exposure to infrared radiation (IR) (Figure 1) [4]. The fusing agent absorbs IR and the subsequent increase in thermal energy sinters the polymer powder together. On the other hand, the detailing agent inhibits powder fusion, which enables precise control over the geometrical outline of the printed part. After the first layer of powder has been sintered, the next layer of material is deposited, and the process repeats until part fabrication is complete. Although comparable to selective laser sintering (SLS), the MJF processes use planar IR exposure to achieve powder fusion instead of a point-wise scanning algorithm.

Although it is a relatively new AM approach, several studies have been undertaken to investigate the properties and applications of MJF-fabricated PA12 parts [5,6,7,8,9,10,11]. There are reports suggesting an absence of significant anisotropy in tensile strength and modulus regardless the print orientation [5]. However, the authors observed that flexural strength and flexural modulus had both varied by up to 30% depending on the print orientation. Elongation at break had also varied by up to 40%, with horizontally built specimens recording the lowest values. In a separate study, no significant anisotropy was also found when comparing horizontally and vertically built specimens [9]. However, contrary to reports in [5], these authors had reported a 20% higher elongation at break for horizontal specimens when compared to vertical ones. In a third study, it was reported that vertically built specimens had displayed slightly higher tensile strength and tensile modulus, but lower elongation at break, when compared to horizontally built specimens [10]. It had been proposed that the additional material weight with increasing part vertical height had led to a denser, less porous part; therefore, to improvements in some mechanical properties of vertical specimens [5,10].

The design freedom enabled by AM technologies presents an opportunity to optimise part designs based on finite element analysis. However, material models based on the intrinsic properties of bulk materials will not be applicable as the AM process imparts physical features that will influence printed part properties; material models specific to both AM technique and material are needed. For example, [12] developed a numerical model describing the ductile behaviour of selectively sintered PA12, while [13] fitted experimental data to the Chaboche model with the Gurson–Tvergaard–Needleman damage model to describe the tensile behaviour of the same material. However, the literature on finite element modelling of MJF-fabricated PA12 is lacking. Based on tensile stress–strain data, MJF-fabricated PA12 had been modelled in the commercial finite element analysis (FEA) package—Abaqus [6]. The authors used an elastic–plastic with isotropic hardening (EPI) material model to describe the stress–strain behaviour of the material. While the authors have validated the material model in compression simulations, it was observed that the material model had overestimated the densification strain and the energy absorption efficiencies. To accurately simulate more complex loading conditions, such as bending, a material model capable of describing both the tensile and compressive behaviour of MJF-fabricated PA12 is needed.

With the general disagreement over the influence of print-orientation on the mechanical properties of MJF-fabricated PA12 in the literature, a more comprehensive study becomes necessary to understand how print-orientation affects said properties. We aim to investigate the influence of print-orientation on the tensile and compressive mechanical properties of MJF-fabricated PA12. The objectives are (i) to characterise the tensile and compressive mechanical properties of MJF-fabricated PA12 at various print-orientations; (ii) to propose a mechanism behind the anisotropy observed in said properties; and (iii) to propose a suitable material model for the finite element analysis of MJF-fabricated PA12.

## 2. Materials and Methods

### 2.1. Materials

PA12 powder (commercial name: HP 3D High Reusability PA12) was provided by HP. It is reported to have the following properties: particle size of 60 μm, melting point of 187 °C, and print density of 1.01 g/cm^3^.

### 2.2. Characterisation Techniques

Mechanical property testing was conducted with an Instron Universal Test Instrument, Model 5900R, with 30 kN load cell. Uni-axial tensile testing was performed using Type-I dumbbell-shaped test bars in accordance with ASTM D638-97. The Instron model XL (long travel) extensometer with a gauge length of 50 mm was used for strain measurement. The Young’s modulus (*E*_Tensile_) and tensile strength at yield (σ_Tensile_) were evaluated at a cross-head speed of 5 mm/min at ambient temperature. *E*_Tensile_ was determined from the slope of the stress–strain data at up to 1% strain. The results presented are an average of three measurements.

Uni-axial compression testing was performed with prism specimens of 12.7 × 12.7 × 50.8 mm^3^ for modulus measurement in accordance with ASTM D695-15. The compressive modulus (*E*_Compressive_) and compressive strength (σ_Compressive_) were evaluated at a cross-head speed of 1.3 mm/min at ambient temperature. *E*_Compressive_ was determined from the slope of the stress–strain data at up to 1% strain. Results presented are an average of three measurements.

The flexural test was performed with a three-point bending test jig using specimens with rectangular cross-section of 12.7 × 4.0 mm and span of 127.0 mm in accordance with ASTM D790-17. The distance between the supports is 50.88 mm. The flexural modulus and flexural strength were evaluated at a cross-head speed of 0.98 mm/min, for a strain rate of 0.1 mm/mm/min, at ambient temperature. Flexural modulus was determined from the slope of the stress–strain data at up to 1% strain.

### 2.3. Test Specimen Preparation

All test specimens were fabricated using an HP Jet Fusion 3D 4200 printer. The available build volume of the printer is 380 × 284 × 380 mm^3^. The “balanced” print mode—which was designed to allow a good compromise between look and feel, dimensional accuracy and mechanical properties—was used. All parts were centred in the build volume, with a space of 10 to 20 mm separating each part to allow complete thermal flow. Parts were fabricated using PA12 powder (80% new/20% old refresh ratio) sourced from HP, along with their proprietary fusing and detailing agents. After printing, the parts were allowed cool in the powder bed for 16 h before being unpacked. The loose powder was recycled, and the printed specimens were cleaned with bead blasting and air blasting. Henceforth, the specimens will be referred to as AM PA12.

**Figure 2:**
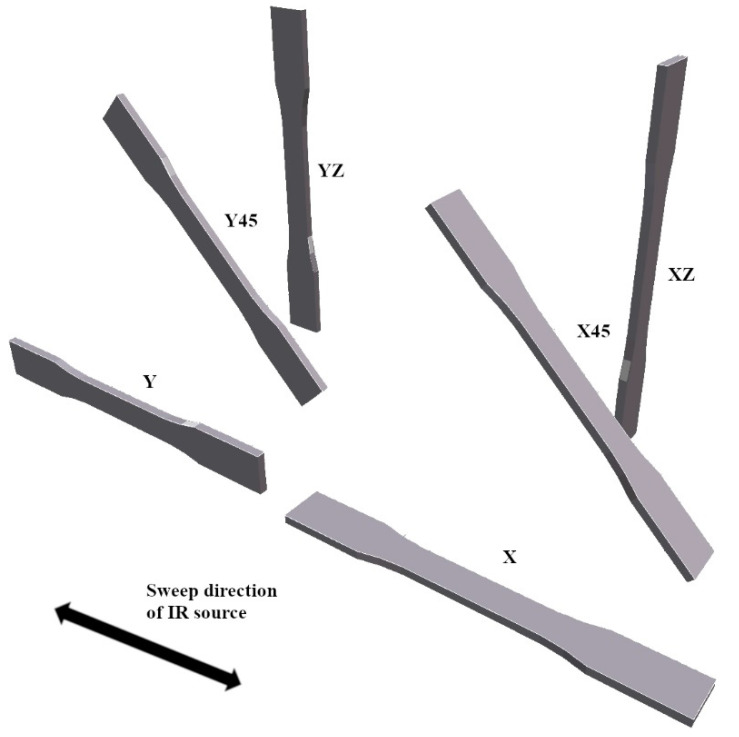
Schematic of the various print orientations with respect to the sweep direction of the IR source.

The specimens and their respective designations according to print orientation are presented in Figure 2. The length of horizontal specimens (X and Y), and therefore the direction of the tensile and compressive testing loads, have been aligned to the sweep direction of the IR source. Compression test specimens have a square cross-section. Therefore, they are identical when printed in the X and Y directions.

### 2.4. Finite Element Analysis

A selection of plasticity models had been evaluated to identify the most suitable material model for polyamide-11 (PA11) fabricated via MJF [14]. Based on their coefficient of determination (R^2^), combined hardening was selected as the most suitable plasticity model for MJF-fabricated PA11. Similar to MJF-fabricated PA11, the tensile stress–strain curve of AM PA12 can be idealised as an elastic, power hardening model [15]. As such, PA12 was modelled using the elastic–plastic with combined hardening material (EPC) model. The combined hardening model consists of a nonlinear kinematic hardening component, which describes the translation of the yield surface in stress through the backstress (*α*); and an isotropic hardening component which describes the change in equivalent stress defining the size of the yield surface ($\sigma^{{}^{\circ}}$) [16]. The constitutive equations that define the EPC model have been comprehensively reviewed and discussed in the literature [17,18] and they are only briefly described here.

The nonlinear kinematic hardening component is defined to be an additive combination of a purely kinematic term (linear Ziegler hardening law) and a relaxation term which introduces non-linearity. The hardening laws for each backstress are:

(1) $${\dot{\alpha }}_{k}=C_{k}\frac{1}{\sigma^{{}^{\circ}}}\left(\sigma -\alpha \right){\dot{\bar{\varepsilon }}}^{pl}-\gamma_{k}\alpha_{k}{\dot{\bar{\varepsilon }}}^{pl}$$

and the overall backstress is computed from the relation:

(2) $$\alpha =\overset{N}{\underset{k=1}{\sum }}\alpha_{k}$$

where $C_{k}$ are the initial kinematic hardening moduli, $\gamma_{k}\;$ refers to the rate at which $C_{k}$ decreases with increasing plastic deformation, and ${\dot{\bar{\varepsilon }}}^{pl}$ is the equivalent plastic strain rate. In this work, $C_{k}$ and $\gamma_{k}$ for five backstress terms were defined as parameters that have been obtained by calibration with experimental tensile and compressive stress–strain data.

Calibration was performed with the Nelder-Mead method [19,20]. The material model parameters were fitted to the experimental data based on the normalised mean absolute deviation. Parametric optimisation was performed with a maximum of 1000 iterations.

**Figure 3:**
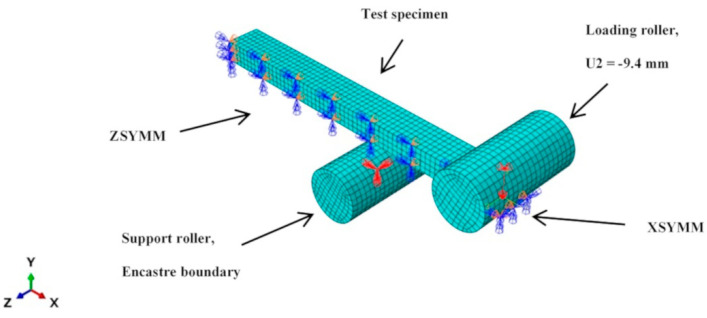
FEA model of flexural bending test set-up.

The calibrated material model was validated with a simulation of three-point bending test (Figure 3). The test specimen was modelled as 3D deformable solid using C3D20 elements. Only one quarter of the test specimen was modelled with symmetry across the XY (ZSYMM) and YZ (XSYMM) planes to reduce computational cost. Meshing was performed with a global seed size of 1 for a total of 1512 elements. The supports of the three-point bending test jig were modelled as 3D discrete rigid using R3D4 elements. Encastre (all DOF removed) boundary condition was applied on the support roller. U2 displacement of −9.4 mm was applied on the loading roller. Contact between the rollers and test specimen was modelled with a friction coefficient of 0.4.

## 3. Results and Discussion

### 3.1. Mechanical Properties

**Figure 4:**
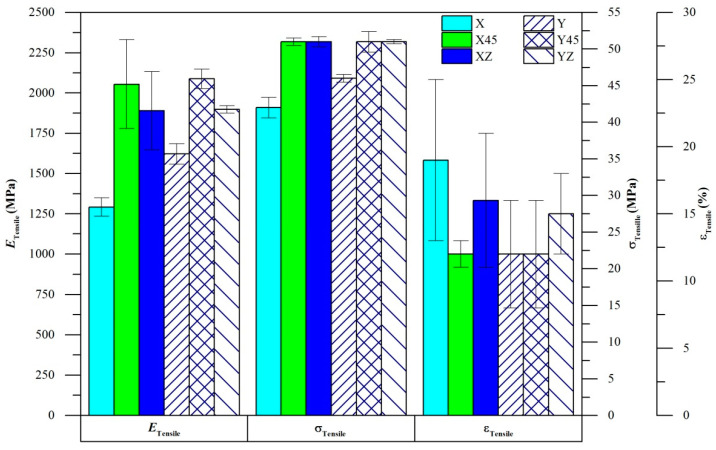
Tensile mechanical properties of AM PA12.

The mechanical properties of AM PA12 were experimentally evaluated in tension and compression. We observe significant print orientation-dependent anisotropy in both tension and compression (Figure 4). In tension, specimens printed in the X-direction had the lowest values of *E*_Tensile_ and σ_Tensile_. Compared to the highest recorded values (Y45-direction), X specimens were approximately 40% and 20% lower in *E*_Tensile_ and σ_Tensile_, respectively. Y specimens had slightly higher values of *E*_Tensile_ and σ_Tensile_ when compared to X-direction specimens. X45 and Y45 specimens had similar tensile properties and had displayed the highest values of *E*_Tensile_ and σ_Tensile_ among the different orientations. XZ and YZ specimens also had similar tensile properties, with approximately 10% lower *E*_Tensile_ than X45/Y45 specimens. X45, Y45, XZ, and YZ specimens had displayed similar values of σ_Tensile_. X specimens have generally displayed the highest ε_Tensile_ among the different orientations. Among the different studies in the literature, the overall trends in orientation-dependent tensile mechanical properties observed in this work most closely resembles the work by Riedelbauch et al. [10].

**Figure 5:**
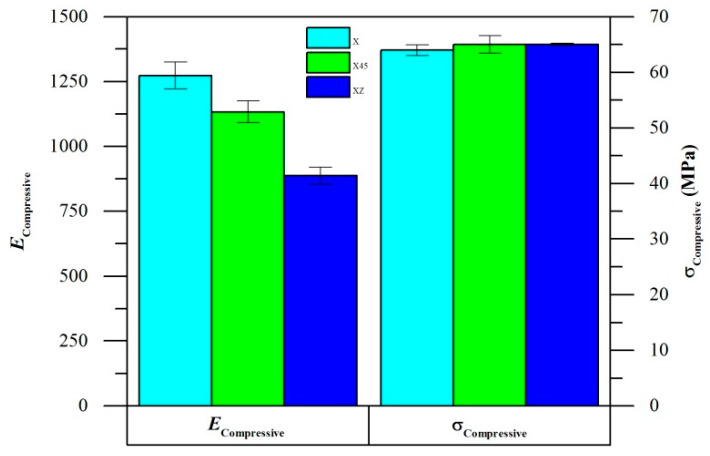
Compressive mechanical properties of AM PA12.

X specimens had the highest *E*_Compressive_, followed by X45 and XZ specimens (Figure 5). All three specimens displayed similar values of σ_compressive_. However, the compression tests were stopped due to buckling failures of the specimens rather than yielding, thus the test may not truly reflect the actual σ_compressive_ limits.

It is a noteworthy observation that while aligning the print orientation of SLS parts to the direction of the tensile loads generally led to improved *E*_Tensile_ and σ_Tensile_ [21,22,23], AM PA12 specimens in the horizontal specimens had generally displayed a lower *E*_Tensile_ but a higher *E*_Compressive_, with X45/Y45 specimens displaying the highest *E*_Tensile_. Within the X45-Y45 and XZ-YZ pairs, similar values of *E*_Tensile_, σ_Tensile_, ε_Tensile_ were observed. On the other hand, distinct differences were observed within the X–Y pair. These observations highlight that after accounting for the alignment of the sweep direction of the IR source to the load direction, there is a benefit in orientating parts towards the vertical print direction for improved tensile mechanical properties. It had been proposed that the compressive effect that the weight of layers in the Z-direction has on prior layers will lead to a denser and less porous structure, which may explain improvements to flexural properties [5]. However, the experimental data in this work showed otherwise, suggesting that a different mechanism may be introducing anisotropy in the mechanical properties of AM PA12.

It should be recognised that despite a printing methodology that resembles conventional powder bed fusion processes such as SLS, the polymer powder in MJF is not melted or sintered directly by the heat source. Instead, sintering in MJF is initiated by the IR-absorption of the fusing agent. As such, it is proposed that building in the vertical directions allowed greater exposure to IR due to the increase in the number of sweeps by the IR source. The thesis is supported by similar observations in parts fabricated by the digital light processing (DLP) technique [24]. The authors observed that an increase in UV exposure time had introduced anisotropy in *E*_Tensile_ and σ_Tensile_, with vertically orientated specimens (equivalent to XZ in this work) displaying greater values than horizontally oriented specimens (equivalent to X in this work). However, following a post-curing process, the anisotropy in mechanical properties between the parts was significantly reduced and became statistically insignificant. Likewise, the increased exposure to IR would have promoted more complete powder fusion in MJF parts.

In this work, the incline of the X45/Y45 parts would have increased the IR exposure on earlier deposited layers that would have otherwise been blocked by increasing part thickness. As such, *E*_Tensile_ varied according to part orientation in the following order: X45/Y45 > XZ/YZ > Y > X. However, printing vertically oriented parts (XZ/YZ) may have resulted in a lower overall part density [21]. As such, while there is an increased fusion between the polymer powders, the porosity within vertically printed specimens would have allowed the part to deform more easily during compression. *E*_Compressive_ varied according to print orientation in the following order: X > X45 > XZ.

### 3.2. Finite Element Model

**Figure 6:**
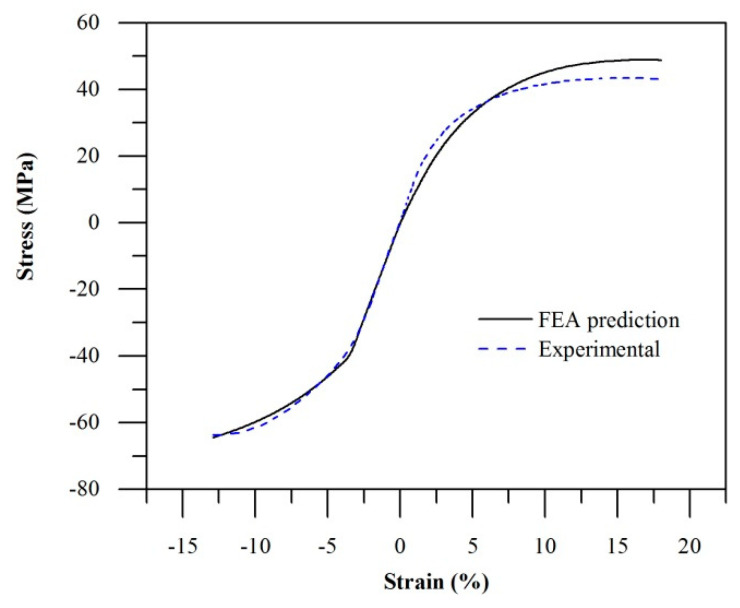
EPC material model predictions compared to experimental stress–strain curve (X-direction).

Based on the compressive stress–strain curve of AM PA12, the EPI material model that was applied in [6] will not be able to accurately represent the material behaviour across the entire compressive and tensile strain range. This is because the EPI material model effectively assumes that the compressive stress–strain curve is identical to that of the tensile stress–strain curve, albeit in terms of compressive stress and strain. The stress–strain behaviour of AM PA12 was therefore modelled using the elastic–plastic with combined hardening (EPC) material model available in Abaqus. Material model parameters were calibrated against experimental tensile and compressive test data for specimens printed in the X-direction only. The FEA predictions are in good agreement with experimental results with a coefficient of determination (R^2^) of 0.94 (Figure 6).

**Figure 7:**
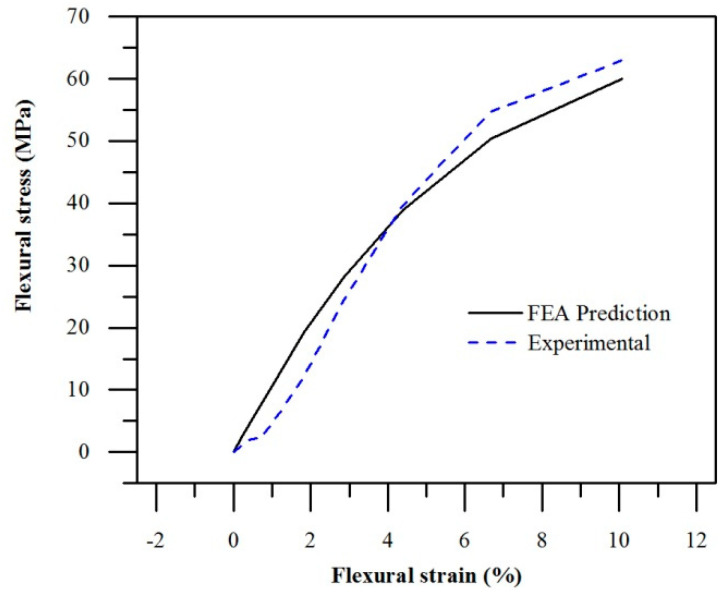
EPC material model predictions compared to experimental three-point bending stress–strain curve (X-direction).

To validate the material model, a simulation of a three-point bending test was performed and compared against experimental data. We observe that the simulations are in good agreement with the experimental results. Although the FEA approach underestimated flexural modulus by 14%, predicted flexural strength only differed by 5% (Figure 7). While the EPC model is unable to capture anisotropy in mechanical properties, as well as strain-rate dependent properties, it was demonstrated that both tensile and compressive stress–strain data should be used to calibrate a robust FEA material model of PA12.

## 4. Conclusions

AM PA12 was characterised in terms of its tensile and compressive mechanical properties. After accounting for the alignment of IR source sweep direction to the test load, we observed significant anisotropy with specimens orientated towards the vertical axis during printing, favouring a higher *E*_Tensile_ while reducing *E*_Compressive_. We attribute the observations to more complete polymer powder fusion as a result of the increased number of sweeps by the IR source when said specimens were printed. Due to significant differences between the compressive and tensile stress–strain behaviour of AM PA12, the EPC material model is a more suitable material model for the FEA of AM PA12 when compared to the EPI material model, which had been applied to model AM PA12 in the literature. The results of three-point bending test simulation is in good agreement with the experimental data. Although the EPC material model is an improvement over the EPI material model for FEA of AM PA12 part designs, more advanced material models may be necessary to capture strain-rate effects and anisotropy of AM PA12.

## Data Availability

Not applicable.
